# Therapeutic properties of a vector carrying the HSV thymidine kinase and GM-CSF genes and delivered as a complex with a cationic copolymer

**DOI:** 10.1186/s12967-015-0433-0

**Published:** 2015-03-04

**Authors:** Irina V Alekseenko, Eugene V Snezhkov, Igor P Chernov, Victor V Pleshkan, Victor K Potapov, Alexander V Sass, Galina S Monastyrskaya, Eugene P Kopantzev, Tatyana V Vinogradova, Yuri V Khramtsov, Alexey V Ulasov, Andrey A Rosenkranz, Alexander S Sobolev, Olga A Bezborodova, Anna D Plyutinskaya, Elena R Nemtsova, Raisa I Yakubovskaya, Eugene D Sverdlov

**Affiliations:** Shemyakin-Ovchinnikov Institute of Bioorganic Chemistry, Russian Academy of Sciences, ul. Miklukho-Maklaya 16/10 Moscow, 117997 Russia; Institute of Molecular Genetics, Russian Academy of Sciences, Kurchatov Sq. 2, Moscow, 123182 Russia; Institute of Gene Biology, Russian Academy of Sciences, ul. Vavilova, 34/5 Moscow, 119334 Russia; Moscow State University, Biological Faculty, ul. Leninskiye Gory, 1–12 Moscow, 119234 Russia; Moscow Hertsen Research Institute of Oncology, Russian Ministry of Health Care, 2nd Botkinskiy proezd 3, Moscow, 125284 Russia

**Keywords:** Cancer suicide gene therapy, Targeted therapy, Preclinical trials, Metastases, Nonviral vectors, HSVtk, PEG-PEI cationic copolymer, GM-CSF, CMV promoter, Internal ribosome entry site

## Abstract

**Background:**

Gene-directed enzyme prodrug therapy (GDEPT) represents a technology to improve drug selectivity for cancer cells. It consists of delivery into tumor cells of a suicide gene responsible for *in situ* conversion of a prodrug into cytotoxic metabolites. Major limitations of GDEPT that hinder its clinical application include inefficient delivery into cancer cells and poor prodrug activation by suicide enzymes. We tried to overcome these constraints through a combination of suicide gene therapy with immunomodulating therapy. Viral vectors dominate in present-day GDEPT clinical trials due to efficient transfection and production of therapeutic genes. However, safety concerns associated with severe immune and inflammatory responses as well as high cost of the production of therapeutic viruses can limit therapeutic use of virus-based therapeutics. We tried to overcome this problem by using a simple nonviral delivery system.

**Methods:**

We studied the antitumor efficacy of a PEI (polyethylenimine)-PEG (polyethylene glycol) copolymer carrying the HSVtk gene combined in one vector with granulocyte–macrophage colony-stimulating factor (GM-CSF) cDNA. The system HSVtk-GM-CSF/PEI-PEG was tested *in vitro* in various mouse and human cell lines, *ex vivo* and *in vivo* using mouse models.

**Results:**

We showed that the HSVtk-GM-CSF/PEI-PEG system effectively inhibited the growth of transplanted human and mouse tumors, suppressed metastasis and increased animal lifespan.

**Conclusions:**

We demonstrated that appreciable tumor shrinkage and metastasis inhibition could be achieved with a simple and low toxic chemical carrier – a PEI-PEG copolymer. Our data indicate that combined suicide and cytokine gene therapy may provide a powerful approach for the treatment of solid tumors and their metastases.

**Electronic supplementary material:**

The online version of this article (doi:10.1186/s12967-015-0433-0) contains supplementary material, which is available to authorized users.

## Background

Chemotherapy, radiotherapy and surgery are conventional treatments for cancer (for recent review, see [1,2]). However, chemotherapy and radiotherapy agents are highly hazardous and, in addition, a majority of cancers have become resistant to current therapeutic options [1,2]. This makes the development of more efficient strategies highly desirable. Various inter- and intracellular barriers to tumor-targeted chemotherapeutic drugs have been considered in detail [3-6].

Great attention was also paid to a strategy of anticancer therapy aimed to potentiate antitumor activity of the immune system [7-12], that looks highly promising.

More than 15 years ago the anticancer armamentarium was extended with gene therapy approaches, the development of which has dramatically accelerated in the recent years (see [13], and for recent reviews [1,2,14-16]). Many approaches to cancer gene therapy have been proposed, and viral and nonviral vectors have been explored. Various strategies of gene therapy have been described (for most recent reviews, see [1,2]. One of them (the suicide gene/prodrug approach or gene directed enzyme prodrug gene therapy, GDEPT) [1,2,14,17-19] is targeted at the systems common to all cancer cells, usually at the replication system. In this regard, this approach resembles chemotherapy with its universal applicability to different types of cancer. However, suicide gene therapy hits its targets from within the cancer cell and is therefore expected to be less toxic to normal cells than classic chemotherapy. GDEPT (other synonyms of this technology see in [19]) is based on the delivery into cancer cells of expressible genes encoding enzymes that can metabolize a separately administered nontoxic prodrug into a cytotoxin. This makes the system much safer than any other known tumor- targeting systems. The generated cytotoxin can not only kill the cancer cell where it was produced but also diffuse into neighboring cells and kill them. This is a so called bystander effect [20-23]. Bystander cell killing may greatly increase the efficiency of GDEPT.

In general, GDEPT looks essentially more powerful than molecular targeted approaches, and recent reports on pre-clinical cancer models demonstrated a high potential of this strategy [1]. Several enzyme-prodrug systems are now in preclinical and clinical trials [1], including the most extensively studied systems of the herpes simplex virus thymidine kinase gene (HSVtk) with ganciclovir (GCV) as a prodrug, and the cytosine deaminase gene (*CD*) of *Escherichia coli* or yeast which converts the non-toxic antifungal agent 5-fluorocytosine (5-FC) into toxic 5-fluorouracil (5-FU).

Major limitations of the suicide gene therapy that hinder its clinical application include inefficient delivery to cancer cells (it is also the problem for chemotherapeutic targeting) and poor prodrug activation by suicide enzymes [14]. Some efforts have been and are currently being pursued to increase the activity of individual suicide enzymes towards their respective prodrugs ([24] and refs. therein).

Another way to overcome these constraints might be to combine suicide gene therapy with immunomodulating therapy. Many cytokines activate the immune system, including interleukins (IL) 2, 4, 7, 12 and 18, interferon γ (IFN-γ), tumor necrosis factor α (TNF-α), and granulocyte–macrophage colony-stimulating factor (GM-CSF), which are among the most potent inducers of anti-tumor activity in a variety of preclinical studies [25-27]. However, it was reported that effects of cytokines are contradictory and depend on the tumor type and disease stage. Moreover, some cytokines can facilitate malignization of tumors and metastasizing [28,29]. Therefore, the choice of cytokines for gene-therapeutic purposes should be based on a thorough analysis of their tests as antitumor agents. Such an analysis indicated that one of the promising candidates was GM-CSF. A comparison of various cytokines showed that GM-CSF enhanced most types of immune responses [30].

It is important that recombinant GM-CSF (Sargramostim) has been extensively used in cancer patients, and its safety is thus well established [31]. Several species of oncolytic viruses were armed with GM-CSF and tested in clinical trials. The trials supported antitumor efficacy of GM-CSF and tumor-specific immune activation [26,31-35]. GM-CSF-secreting vaccines for solid tumors demonstrated promising evidence of safety in early phase clinical testing [36,37]. An oncolytic adenovirus coexpressing IL-12 and GM-CSF in combination with vaccination demonstrated synergistic antitumor effects [38].

A combination of *HSVtk* in an adenoviral vector with another vector carrying both the *GM-CS*F and *IL-2* genes was tested. The results obtained demonstrated that coexpression of GM-CSF and IL-2 could enhance the effect of HSVtk suicide gene therapy [39]. Other studies also confirmed the efficacy of using IL-2 and GM-CSF in combination with HSVtk in adenoviral vectors [40]. Also, a combination of GM-CSF and HSVtk gene therapy showed a greater therapeutic effect than HSVtk alone [41,42].

However, there is some uncertainty about the use of GM-CSF as an agent for the induction of antitumor immunity [43-45]. GM-CSF may play a key role in the appearance of host immune cells with a suppressive phenotype that poses a significant problem to successful therapy for metastatic cancers [46-48]. To explain the successful antitumor role of GM-CSF in combination with suicide genes, it was suggested that when tumor cells are destroyed and release tumor-specific antigens, GM-CSF in the tumor microenvironment increases antigen uptake and presentation by antigen presenting cells [31,41].

This hypothesis is in line with the efficient antitumor activity of oncolytic viruses armed with GM-CSF [26,32,49-54]. Therefore, a combination of cancer cell destroying agents with GM-CSF can be a powerful tool for killing cancer and metastasis cells. Up to now, all such combinations used viral vectors that have some limitations in their application to clinical practice [2,55].

To our knowledge, the present study is the first one to test the efficacy of an HSVtk/GM-CSF combination in one vector using a nonviral system (a PEG-PEI copolymer) for its delivery. Nonviral vectors are advantageous over viral vectors due to low immunogenicity, practically unlimited packaging capacity for genetic material, as well as simple and low-cost production [56]. They are used as components of self-assembling complexes with anionic DNA (‘polyplexes’). Among cationic polymers, polyethylenimine (PEI) attracts special attention and is the most intensively studied polymer for gene-therapy purposes. The properties and behavior of PEI-containing polyplexes, modified with polyethylene glycol (PEG) and the cell-penetrating peptide TAT, in different cell lines can be adjusted to achieve higher transfection efficiency [57].

Here, the system was tested *in vitro* in various cell lines, *ex vivo*, and *in vivo* on mouse models, and promising results were obtained.

## Materials and methods

### Construction of expression plasmids

**Figure 1:**
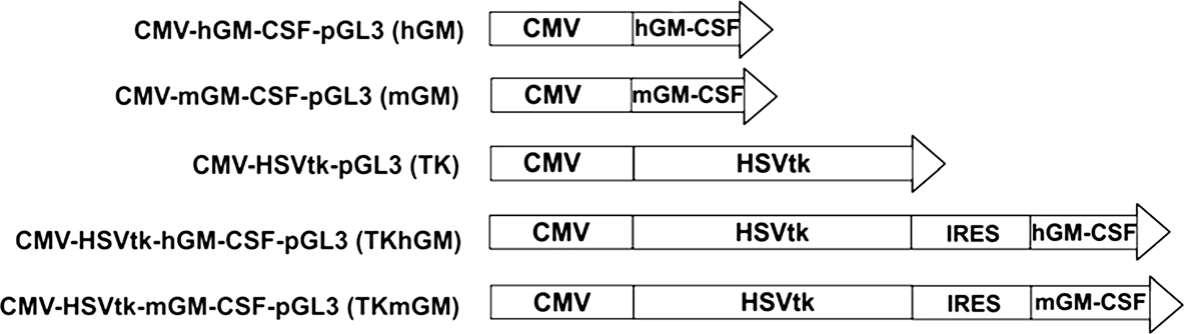
**Schematic representation of the expression constructs used.** On the left – construct names. The SV40 polyA signal is omitted from the schemes. CMV – major immediate-early promoter of human cytomegalovirus; hGM-CSF and mGM-CSF – human and mouse genes of granulocyte macrophage colony stimulating factor, respectively; HSVtk –herpes simplex virus thymidine kinase gene, IRES - internal ribosome entry site of encephalomyocarditis virus.

The cDNA of the hGM-CSF gene was amplified from plasmid hGM-CSF-pBK, (kindly provided by S. Larin, IGB RAS, Moscow) using primers 5′-TTATCGATATGTGGCTGCAGAGC and 5′-TTGGATCCTCACTCCTGGACTGG that had at their 5′-ends the restriction sites of ClaI and BamHI, respectively. The amplificate was ligated into pAL-TA vector (Evrogen, Moscow, Russia) containing an SV40 polyA fragment. The hGM-CSF-polyA sequence was excised with ClaI and SphI and cloned into retroviral vector pFB-neo (Stratagene, La Jolla, USA) that contained a picornavirus internal ribosome entry site (IRES) and was hydrolyzed by these restriction endonucleases. The IRES-hGM-CSF-polyA cassette was excised from this vector with NotI and BamHI, and its ends were filled in with Klenow fragment. After this, the cassette was blunt-end ligated into CMV-HSVtk-pGL3 vector [58], split at a unique site by XbaI and treated with Klenow fragment. This gave the construct CMV-HSVtk-hGM-CSF-pGL3 (designated as TKhGM, Figure 1) harboring the HSVtk and hGM-CSF genes under the control of the CMV promoter.

To obtain the construct CMV-HSVtk-mGM-CSF-pGL3 (designated as TKmGM, Figure 1), the mGM-CSF cDNA was amplified with primers 5′-TTATCGATATGTGGCTGCAGAAT and 5′-TTGGATCCTCATTTTTGGCC that had at their 5′-ends the restriction sites of ClaI and BamHI, respectively. The amplified fragment was cloned into the construct CMV-HSVtk-hGM-CSF-pGL3 split with ClaI and BamHI.

The design of such constructions was reported earlier [59].

### Cell cultures

The following cancer cell lines were used: HEK293 (transformed human kidney cells), HT1080 (human fibrosarcoma), A431 (human epidermoid carcinoma), and Calu1 (human epidermoid lung carcinoma) from ECACC (Salisbury, UK), and C26 (murine colon adenocarcinoma) from Cell Lines Service (CLS GmbH, Eppelheim, Germany).

Sarcoma 37, Lewis lung carcinoma (LLC), and cervical squamous carcinoma (CSC5) mouse tumors were obtained from the Department of Tumor Strains of Blokhin RRCO RAMS (Moscow, Russia).

Tumor cell lines LLC, CSC5 и S37 were obtained by culturing dissociated cells from the corresponding transplantable mouse tumors in RPMI 1640 medium.

S37, CSC5 and C26 cell lines were grown in RPMI1640 medium containing 12.5% fetal calf serum, 60 μg/ml penicillin, 100 μg/ml streptomycin, and 0.25 μg/ml amphotericin at 37°C and 5% CO_2_.

All other cells were grown in DMEM/F12 medium containing 10% fetal calf serum, 60 μg/ml penicillin, 100 μg/ml streptomycin, and 0.25 μg/ml amphotericin at 37°C and 5% CO_2_.

All materials for cell culturing were obtained from Invitrogen (Carlsbad, CA, USA).

### DNA transfection

#### Lipofection

For transfection, cells were seeded into 24-well plates or 25-см^3^ flasks and incubated in serum-containing medium for 24 h. Cells were transfected with Lipofectamine 2000 (Invitrogen, Carlsbad, CA) according to the manufacturer’s recommendations. After this, the transfected cells were cultured for 48 h at 37°С and 5% CO_2_.

For the GM-CSF protein quantification using ELISA and Western-blot analysis, the transfection was performed in 25-cm^3^ flasks for 48 h.

#### Transfection with a PEI-PEG-TAT copolymer

A polyethylenimine (PEI)-polyethylene glycol (PEG)-TAT peptide copolymer (PPT) was obtained as described previously [57]. In this study, the ratio of PEI to PEG in polyplexes was optimized according to [57] to achieve maximum transfection efficiency. A DNA-PPT complex was obtained by vigorous mixing of one volume of a PEI-PEG-TAT copolymer in 0.1 M borate (pH 7.5), one volume of 20% glucose, 20 мМ HEPES buffer (pH 7.4), and two volumes of plasmid DNA water solution (160 μg/ml DNA). The resulting solution contained 80 μg/ml DNA and 12.8 μg/ml copolymer. The mixture was incubated for 1–2 h at room temperature and used for injection into animals in *in vivo* experiments. Cells were transfected in 24-well plates with the resulting solution 20-40-fold diluted (depending on the cell line) with growth medium and added to wells in a volume of 1 mL per well. The transfected cells were cultured for 72 h at 37°С and 5% CO_2_, and further incubated for 24 h at 37°С and 5% CO_2_ after addition of an equal volume of nutrient medium.

All plasmids used for transfection were isolated using an EndoFree Plasmid Maxi Kit (Qiagen, Valencia, CA).

### Measurement of transfection efficiency

To determine transfection efficiency, reporter plasmids carrying the EGFP reporter gene driven by the pCMV immediate early promoter were used. Cells were transfected in 24-well plates with Lipofectamine 2000 (LFA) or PEI-PEG-TAT. In 48 h after transfection with LFA or 72 h in the case of PPT, cells were photographed on a Nikon fluorescence microscope (200 × field) at 395-nm excitation, and the number of fluorescent cells was determined in 10 microscopic fields. Cells were further washed with PBS buffer, detached from the plate surface with trypsin, and suspended in PBS at 10^6^ cells per 1 ml. The percentage of transfected cells was determined using a fluorescence-activated cell sorter FACS Scan Analyzer (BD Bioscience, San-Jose, CA). The data were processed using BD CellQuest Pro (BD Bioscience, San-Jose, CA) and WinMDI 2.8 (by Joe Trotter) software. The transfection efficiency was determined as the percentage of cells whose fluorescence intensity exceeded that of non-transfected cells.

### *In vitro* cell sensitivity to ganciclovir

Transfected cells were detached from the plate surface, seeded into 96-well plate at 1500-2000 cells per well and incubated for 12–18 h at 37°С and 5% CO_2_. The wells were then supplemented with ganciclovir Cymevene® (F. Hoffman-La Roche Ltd, Switzerland) solution up to final concentrations of 12.5, 50 or 200 μM, and incubated for 48 h, after which the GCV solution was replaced with a fresh one, and cells were additionally incubated for 48 h. The number of viable cells was determined by the MTS assay according to the protocol (CellTiter 96® Aqueous One Solution Cell Proliferation Assay, Promega, Madison, WI). The results were expressed as a ratio between the number of viable cells in the plates that contained the drug and their number in the corresponding drug-free controls. Three independent transfections were performed in each experiment.

### HSVtk and GM-CSF production by transfected cells

The transfected cells were lyzed in SDS sample buffer, and proteins were separated on a 10% SDS-PAGE and transferred into PVDF membranes. A goat polyclonal antiserum against HSVtk (Santa Cruz Biotechnology, Santa Cruz, CA) and donkey anti-goat IgG-horseradish peroxidase conjugates (Santa Cruz, CA, USA) were used to visualize thymidine kinase. Detection of reactive bands was facilitated by using a horseradish peroxidase-linked secondary conjugate and ECL detection reagents (Biorad, USA).

mGM-CSF and hGM-CSF produced by transfected cells were measured by ELISA of culture medium using a commercial kit (R&D Systems, Minneapolis, MN).

### Measurement of the biological activity of hGM-CSF

The biological activity of hGM-CSF was determined by the ability of hGM-CSF-containing conditioned medium to maintain proliferation of the hGM-CSF-dependent erythroleukemia cell line TF-1 [60].

### Measurement of the biological activity of mGM-CSF

The biological activity of mGM-CSF was estimated by the ability of GM-CSF-containing conditioned medium to initiate differentiation of mouse bone marrow precursor cells. The precursor cells were cultured in medium supplemented with conditioned medium obtained from S37 cells treated with TKmGM-PPT. As a negative control, we used conditioned medium obtained from S37 cells transfected with a plasmid that carried the HSVtk and mGM-CSF genes without promoter and was complexed with a PPT copolymer. The procedure was performed as described in [61]. The cells were stained with FITC-labeled antibodies (Caltag, Buckingham, UK) specific for F4/80 (a marker of mature macrophages), IA^d^ (an innate activation marker of macrophages and dendritic cells), and Gr-1 (a marker of granulocytes), or with PE (phykoerythrin)-labeled antibodies against CD86 (a marker of mature dendritic cells). The stained cells were cytofluorometrically analyzed for the presence of differentiated cells.

### *Ex vivo* experiments

In *ex vivo* experiments, we used C57Bl/6 mice (Animal Breeding Facility - Branch of Shemyakin & Ovchinnikov Institute of Bioorganic Chemistry, Puschino, Moscow Region, Russia) with an initial medium body weight of 19.0 ± 1.4 g.

LLC cells were transfected with the CMV-HSVtk-pGL3 (designated as TK) and TKmGM constructs, whereas non-transfected cells were used as the control. The transfection was performed for 3 h at 30 μg DNA and 75 μl Lipofectamine 2000 (LFA) per 75-cm^3^ flask. In 24 h after transfection, the transformed and non-transformed LLC cells were detached with 3 ml trypsin per flask, suspended in complete DMEM/F12 medium, washed twice with PBS, and suspended in PBS at 2 × 10^6^ cells/ml. 100-μl aliquots (2 × 10^5^ cells) of the suspension were subcutaneously injected into the right dorsal flank of animals. Starting from day 6 after inoculation and after the appearance of palpable tumors, tumor size was measured using an electronic caliper.

Tumor volume was calculated using the formula А*В^2^/2 [62], where A and B is the length and width of the tumor, respectively. An euthanasia criterion was the tumor size that has reached 2000 mm^3^.

Each test group of animals injected with LLC cells transformed with the TK and TKmGM constructs contained 10 animals. Control groups contained 6 animals and were injected with non-transformed LLC cells. Animals were uniformly divided among the groups according to their weight.

The average size of tumors was calculated as the total tumor volume divided by the number of animals in the group. The measurement was done until the death of the first animal in each group.

Survival rate of mice was determined as the ratio of the number of mice, having no tumor or tumor with a volume less than 2000 mm^3^, to the total number of mice in the group. The calculations were carried out until the death of the last mouse with tumor.

5 mg/ml GCV in PBS buffer (Invitrogene, Carlsbad, CA, USA) was intraperitoneally injected into animals for 10 days, two times a day in a dose of 75 mg/kg, starting from the second day (48 h) after transplantation of carcinoma cells.

### *In vivo* experiments

Six- to eight-week-old F1 (С57Bl/6j × CBA), BDF1, and BALB/c mice were obtained from the Research Centre of Biomedical Technologies, RAS. All animal protocols were performed in accordance with the “Guidelines to Carry out Preclinical Trials of Pharmaceuticals” [62], and the experiments were approved by the Bioethical Committee of the Moscow Hertsen Research Institute of Oncology (MHRIO).

In these experiments we used the TK, mGM and TKmGM constructs complexed with the PEI-PEG-TAT copolymer (designated as TK-PPT, mGM-PPT and TKmGM-РРT, respectively) or with LFA (TK-LFA, mGM-LFA and TKmGM-LFA, respectively). In the case of PPT, the animals were injected with a 80 μg/ml DNA and 12.8 μg/ml copolymer solution. For LFA, the injected solution contained 2.5 μl LFA per 1 μg DNA.

The DNA-PPT/GCV system was injected into animals of the experimental groups at the doses and according to the schedule described in the Results and Discussion section. Control animals were injected with either pure buffer or DNA-PPT and GCV separately at the same doses and according to the same schedule. Each group contained 8–12 animals, and each experiment was repeated in triplicate.

The mouse S37, LLC and cervical squamous carcinoma (CSC5) tumor strains were maintained in syngeneic mice using standard methods. Tumor cells of the 2^nd^- 8^th^ passages were used *in vivo.* The C26 colon adenocarcinoma cells were maintained using standard protocols.

The tumor cells or tissues were subcutaneously (s.c.) injected into mice at the following doses per mouse: Sarcoma 37 (F1 С57Bl/6j × CBA mice) - 2 × 10^6^ cells, С26 (BALB/c mice) - 4 × 10^4^ cells, LLC (BDF1 mice) – 30 mg of tumor tissue, CSC5 (BDF1 mice) - 20 mg of tumor tissue in 0.1 ml of isotonic (0.9%) NaCl solution. Primary tumors were formed in 90-100% of animals. They had standard growth patterns, and S37 also had the ability to form metastases.

***Evaluation of antitumor and antimetastatic efficacy*** was based on measuring the tumor volumes of the primary and metastatic nodes, the lifespan of animals and frequency of lymphogenic metastasis in different groups.

The tumor growth delay (TGD, days) was calculated by the formula:

TGD = TTE_exp_–TTE_cont_, where

TTE_exp_ and TTE_cont_ are the periods of achieving a certain tumor volume in the experimental and control groups, respectively. The reference tumor volume varied for different tumors from 500 mm^3^ for S37 (TGD_500_) to 1000 mm^3^ for CSC5 (TGD_1000_) and 1500 mm^3^ for C26 (TGD_1500_).

Tumor metastasis inhibition (MI,%) was calculated by the formula:

$$ MI=\frac{Vmts(c)- Vmts\left( \exp \right)}{Vmts(c)}*100\% $$_,_ where

*V*_*mts*_*c –* mean volume of metastatic lymph nodes in the control groups,

*V*_*mts*_*exp –* mean volume of metastatic lymph nodes in the experimental group.

Frequency of lymphogenic metastasis (FLM,%) was calculated by the formula:

$$ FLM=\frac{M}{T}*100\% $$_,_ where

M – the number of animals in the group that had metastases in lymph nodes,

T – the total number of animals in the group.

The presence of metastatic tumor cells in the enlarged lymph nodes of tumor bearing mice was proven using histological examination of the dissected tissues. The lymph nodes were dissected at autopsy, measured in three cross dimensions by a caliper, fixed in 10% neutral buffered formalin, and embedded into paraffin. Tissue sections stained by haematoxylin and eosin were examined.

The increase in lifespan (ILS,%) was calculated by the formula:

$$ ILS=\frac{MLS\left( \exp \right)-MLS(c)}{MLS(c)}*100\% $$, where

*MLS(exp) –* mean lifespan in the experimental group (days),

*MLS(c) –* mean lifespan in the control groups (days).

### Statistical analysis

Statistical analysis was performed using Statistical software version 7.0 (StatSoft, Inc.). The statistical significance of differences between groups was estimated using the t-Student test, Fisher’s test and the Mann–Whitney U-criterion depending on the number of observations and the distribution of parameter values in the group. The differences were considered significant at p < 0.05. Survival data were presented as Kaplan-Meier curves.

## Results

### Construction and *in vitro* properties of IRES vectors for coexpression of the HSVtk and GM-CSF genes

We constructed a series of expression plasmids in which the human cytomegalovirus (CMV) major immediate-early promoter directed the expression of the single or tandemly linked *HSVtk* and *GM-CSF* genes. Since the GM-CSF protein is species-specific, and the efficacy of gene therapeutic constructs was tested in tumor-inoculated animals, we prepared two constructs, one of which contained the mouse *gm-csf* gene (mGM-CSF) and was designed for model experiments in mice, whereas another one contained human *GM-CSF* and was designed for testing of toxicity and subsequent clinical trials as well as for comparative analysis of the functional properties of human and mouse GM-CSF containing constructs.

Simultaneous expression of two genes under the control of one promoter was enabled by the insertion of the internal ribosome entry site (IRES) between the genes.

The structure of the vectors constructed is presented in Figure 1. Each vector contained either the single genes or their tandem combination under the control of the CMV promoter.

Transient transfection experiments showed that the expression level of the *HSVtk* and mouse and human *GM-CSF* genes within bicistronic expression constructs obtained was close to that in the constructs containing single genes (Additional file 1: Figure S1).

HEK293 cells transfected with the TKhGM construct produced 110 ng hGM-CSF per 10^6^ cells a day, whereas transfected with the control plasmid CMV-hGM-CSF-pGL3 (designated as hGM) – 115 ng, and transfected with TKmGM and CMV-hGM-CSF-pGL3 (designated as mGM) – 182 and 190 ng mGM-CSF, respectively. Conditioned medium obtained from non-transfected cells did not contain GM-CSF indicating that GM-CSF was produced only due to the expression of the *GM-CSF* gene within the vectors obtained.

Estimations of the HSVtk biological activity revealed that incubation with GCV was equally efficient in killing cells transfected both with TK and TKmGM (Additional file 2: Figure S2).

The human and mouse GM-CSF protein produced by the expression vectors was found to be biologically active (data not shown).

### Use of the PEG-PEI-TAT copolymer for delivery of genetic information into cancer cells: comparison with lipofectamine

Transfection of cell lines *in vitro* was performed using a standard procedure with LFA. However, the use of LFA as part of gene therapeutic systems is limited due to its toxicity and high price.

In this work, we used polyplexes based on polyethylenimine (PEI) conjugated with a heterobifunctional PEG derivative (N-hydroxisuccinimide ester maleimido-PEG-24) to make the complex more hydrophilic. To facilitate penetration into cells, the TAT cell-penetrating peptide (GRKKKRRQRC) was attached to the PEI-PEG copolymer [57,63]. The efficiency of transfection of cancer cells of different origin with PEG-PEI-TAT (PPT) and LFA was compared using a GFP reporter gene. The percentage of cells transfected using PPT depended on the cell type and was in a range of 25-45% for S37 cells, 30-40% for C26, 20-45% for A431, about 20% for HT1080, and 5-30% for LLC cells. For the same cell lines, the transfection level using LFA was 60–75, 45–65, 20–30, 40–55, and about 20%, respectively.

Also, it was shown that in С26, A431 and НT1080 cell lines the cytotoxic effect of the TKhGM-PPT or TKmGM-PPT complex with GCV was weaker than that of TKGM-LFA, whereas in S37 cells the cytotoxic effects of TKGM-PPT and TKGM-LFA were comparable (Additional file 3: Table S1) .

It is important to note that the *in vivo* antitumor efficacies of LFA and PPT deliveries were approximately the same.

Our experiments demonstrated that TGD, the lifespan, MI and FLM were almost equal for TKmGM and TK constructs combined with GCV irrespective of the used delivery system - LFA or PPT (See Additional file 4: Table S2, Additional file 5: Figure S3). The reason of this phenomenon is under investigation, but probably the known enhanced permeability and retention effect [4] is somehow involved.

### *Ex vivo* antitumor efficacy of TK/GCV and TKmGM/GCV

To evaluate the contribution of GM-CSF to the therapeutic potential of the prepared constructs, the antitumor efficacy of the TK and TKmGM construct was measured *ex vivo* in C57BI/6 mice. The mice were subcutaneously inoculated with LLC cells transiently transfected with the TK or TKmGM construct using LFA.

We studied 3 groups of mice: a group inoculated with non-transfected LLC cells (group K), a group inoculated with LLC cells transfected with the TK construct (group TK), and a group inoculated with LLC cells transfected with TKmGM (group TKmGM). Then, half of the animals from each group were intraperitoneally injected with GCV solution at a dose of 75 mg/kg twice a day for 10 days (groups K/GCV, TK/GCV and TKmGM/GCV), whereas the second half were injected with phosphate buffered saline (PBS) as placebo control (groups K/PBS, TK/PBS and TKmGM/PBS).

**Figure 2:**
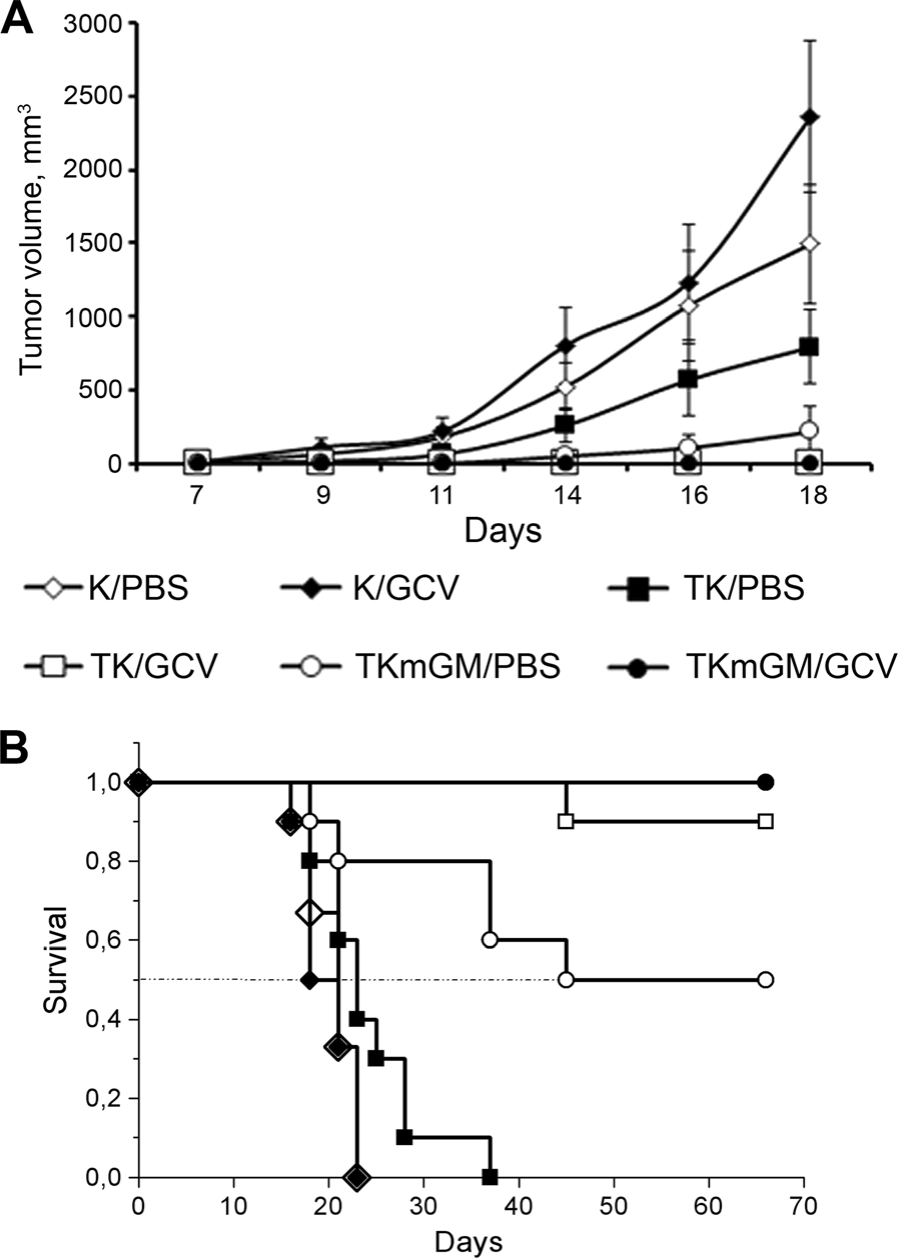
**Effect of** ***ex vivo*** **transformation of LLC cells with the TK and TKmGM constructs combined with administration of GCV on A) tumor growth rate, and B) animal lifespan after transplantation of the transfected cells into C57Bl/6 mice.** The data represent mean values for treatment groups of ten animals and control groups of six animals. We studied 6 groups of mice: two control groups (K/GCV and K/PBS) inoculated with non-transfected cells; two experimental groups (TK/GCV and TK/PBS) inoculated with LLC cells transfected with TK; and two experimental groups (TKmGM/GCV and TKmGM/PBS) inoculated with LLC cells transfected with TKmGM using LFA. The animals of groups К/GCV, ТK/GCV and TKmGM/GCV received intraperitoneal injections of ganciclovir in a dose of 75 mg/kg twice a day for 10 days. The animals of groups К/PBS, ТK/PBS and TKmGM/PBS received phosphate buffered saline (PBS) as a placebo instead of GCV. Starting from day 6 after transplantation, we measured the volume of developed tumors. The euthanasia criterion was the tumor volume that exceeded 2000 mm^3^. A) Tumor volume (in mm^3^, Y-axis) versus time since cell transplantation (X-axis). Mean ± SEM values are shown. B) Survival period of mice after transplantation of the transfected and non-transfected LLC cells.

As seen from Figure 2A, on day 18 of the experiment (the last day when all animals were still alive), the animals inoculated with the TKmGM/GCV or TK/GCV system showed no indications of tumor development. The tumor growth in the group of animals inoculated with the TKmGM/PBS combination was markedly slower than that in the TK/PBS group or control groups, and the difference was statistically significant (p < 0.05). Since the animals inoculated with TKmGM/PBS or TK/PBS received phosphate buffered saline (PBS) instead of GCV, the suppression of tumor growth in the TKmGM/PBS group was most probably due to the presence of the GM-CSF gene. On day 18 of the experiment, the mean tumor volume in the K/GCV control group exceeded that in the K/PBS control group, however, the difference was not statistically significant (p = 0.262).

By the end of the experiment (on day 60), all animals from the TKmGM/GCV group were alive, whereas 10 and 50% of the animals from the TK/GCV and TKmGM/PBS groups, respectively, have died. All mice of the TK/PBS, PBS, and GCV groups died (Figure 2В). All animals that have survived by day 60 of the experiment did not produce recurrent tumors during an one-year period of observation after the end of the experiment. Thus, both the TK and TKmGM constructs demonstrated antitumor effect, although this effect was much more pronounced in the case of TKmGM. This experiment was repeated in triplicate, and in all cases the survival rate in the TKmGM/GCV group was higher than that in the TK/GCV group.

Similar results were obtained earlier in the work of Castleden *et al.*, where the authors in *ex vivo* experiments compared antitumor effects of a construct, in which the *HSVtk* and *GM-CSF* genes shared a common promoter, and of constructs harboring the single HSVtk or GM-CSF genes [64].

Further comparative analysis of the therapeutic potential of the constructs was performed *in vivo* in mice.

### Tumor growth delay and prolongation of the survival period due to TKmGM-PPT/GCV therapy of tumor-bearing mice

We used PEG-PEI-TAT (PPT) copolymers for *in vivo* delivery of the therapeutic constructs obtained [57,65]. We showed that the developed copolymers are non-immunogenic and can be easily modified for targeted delivery (to be published separately).

The selection of the most efficient therapeutic scheme for the copolymer/DNA polyplexes obtained was done using F1 С57Bl/6j x CBA mice subcutaneously inoculated with sarcoma 37 cells. These cells were characterized by the highest level of transfection with the polyplexes.

The most efficient therapeutic scheme for TKmGM-РРТ/GCV was found to be triple intratumoral injection of TKmGM-РРТ at a unit dose of 0.04 μg DNA per 1 mm^3^ tumor volume with 5-day intervals (Table 1А) at the background of GCV injection for 15 days.

**Table 1:** **Effect of intratumoral administration of various complexes on mice inoculated with sarcoma 37**

**A) Scheme of administration**						

**B)**						
**Constructs and controls**	**Lifespan, days**	**ILS,%**	**TGD**_**500mm3,**_ **days**	**FLM,%**	**Volume of lymph nodes, mm** ^**3**^	**MI,%**
TKmGM-PPT/GCV	60 ± 22	70^*^	14.1	33^*^	100 ± 45	82^*^
TK-PPT/GCV	57 ± 15	62^*^	11.6	58^*^	186 ± 80	67^*^
mGM-PPT/GCV	39 ± 13	9	4.1	50^*^	189 ± 91	66
TKmGM-PPT/PBS	41 ± 7	16	3.9	42^*^	298 ± 264	47
TK-PPT/PBS	38 ± 4	8	2.6	75	459 ± 339	18
mGM-PPT/PBS	48 ± 4	35	3.0	58^*^	349 ± 242	38
Control/GCV	40 ± 3	6	0.3	100	457 ± 121	19
Control/PBS	35 ± 3	-		100	562 ± 316	

F1 (С57Bl/6jxCBA) female mice (12 animals in each group) were inoculated with sarcoma 37 on day zero. TKmGM (CMV-HSVtk-mGM-CSF-pGL3 construct), TK (CMV-HSVtk-pGL3), mGM (CMV-mGM-CSF-pGL3); PPT - polyethylenimine-polyethylene glycol-TAT peptide copolymer; PBS – phosphate buffered saline (placebo); GCV – ganciclovir. Control - the group that received only GCV or PBS. Administrations of the complexes are shown by arrows in the scheme. GCV was administered intravenously twice a day with an interval of 12 h in a daily dose of 150 mg/kg for 15 days (total dose 2.25 g/kg). ILS –increase in lifespan, TGD –tumor growth delay, FLM - frequency of lymphogenic metastasis, MI –metastasis process inhibition, mean values. ILS, FLM, MI (compared with the control group of animals that received PBS) and the volume of lymph nodes were measured on day 30 after inoculation. The constructs and control solutions were administered intratumorally with a 5-day interval between administrations. PBS was administered in volumes equivalent to the GCV administration scheme. The PPT concentration in injected solutions of the constructs was 25 μM.

^*^- statistically significant values (p < 0.05).

### Comparison of the antitumor effect of the TK, mGM and TKmGM constructs delivered by PEG-PEI-TAT in mice with sarcoma 37

Mouse sarcoma 37 cells were subcutaneously inoculated into F1 С57Bl/6j x CBA mice. The treatment according to the scheme in Table 1А was started on day 7 of tumor growth, when the mean tumor volume was about 100 mm^3^.

We used polyplex solutions at PPT concentrations in a range of 12.5-25 μM, because we have earlier shown that the results did not depend on the PPT concentration within this range (data not shown).

**Figure 3:**
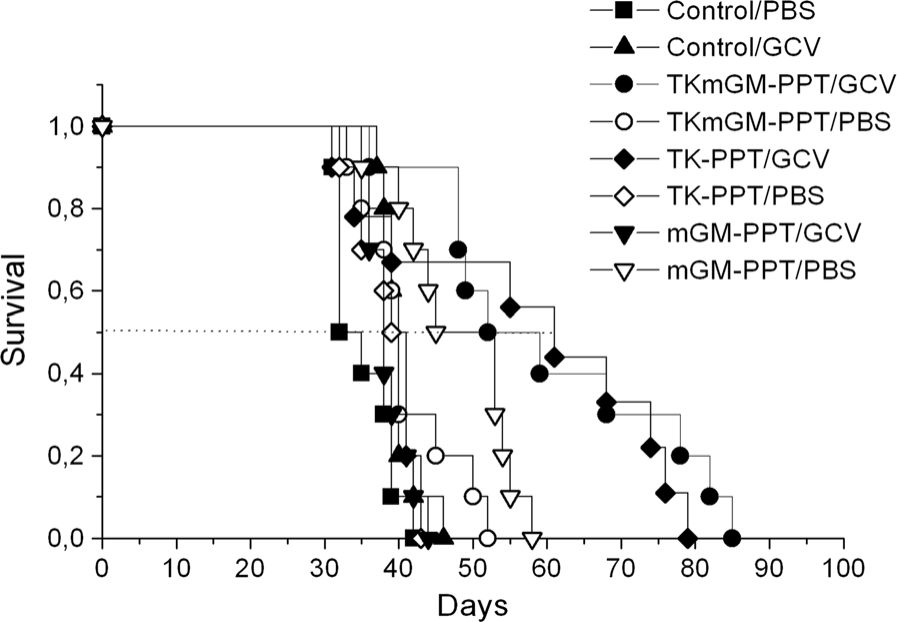
**Survival of S37-bearing mice after injection of TK-PPT, mGM-PPT and TKmGM-PPT with or without ganciclovir.** TKmGM (CMV-HSVtk-mGM-CSF-pGL3 construct), TK (CMV-HSVtk-pGL3), mGM (CMVmGM-CSF-pGL3); PPT - polyethylenimine-polyethylene glycol-TAT peptide copolymer; PBS – phosphate buffered saline (placebo); GCV – ganciclovir.

As seen from Table 1 and Figure 3, intratumoral injections of the TK/GCV or TKmGM/GCV polyplexes had a biologically significant antitumor effect. On day 30, the extension of animal lifespan in mice treated with TKmGM/GCV and TK/GCV was as high as 70 and 62%, respectively. The tumor growth delay (TGD_500_) for the combination TKmGM/GCV was 14.1 days, and for the combination TK/GCV – 11.6 days.

At the same time, injection of the mGM/GCV, TKmGM/PBS, TK/PBS or mGM/PBS combinations without GCV or solely GCV had an insignificant effect on tumor growth and animal lifespan.

### Comparison of the antimetastatic effect of the TK, GM and TKmGM constructs delivered by PEG-PEI-TAT in mice with sarcoma 37

Transplantable mouse sarcoma 37 is characterized by extensive lymphogenic metastasis. According to our observations, popliteal and inguinal lymph nodes are the first to be affected by metastases in a mouse bearing the tumor implanted subcutaneously in a hind paw. The inguinal lymph nodes are preferable for S37 metastasis assessment because the popliteal lymph nodes are often involved into the area of the primary tumor growth.

Progressive growth of S37 tumor in mice was accompanied by an increase in the regional lymph nodes size. The mean volume of the inguinal lymph nodes in normal C57Bl/6j × CBA mice was found to be 16 ± 6 mm^3^. The mean volume of ipsilateral and contralateral lymph nodes measured on day 35 of the tumor growth in mice bearing subcutaneous S37 tumor varied from 350-500 mm^3^ to 120-200 mm^3^, respectively.

The presence of metastatic tumor cells in the enlarged lymph nodes of tumor bearing mice was proven using histological examination of the dissected tissues. The extent of involment of lymph nodes, as a rule, correlated with their size. In the lymph nodes with the volume less than 100 mm^3^ dissected on day 30–35 after tumor inoculation, the area occupied by metastatic cells usually did not exceed 20%. Microfoci of tumor cells or single tumor cells could be revealed (the estimation was made using serial sections). For the nodes with a 100–250 mm^3^ volume, the involved section area varied from 20 to 75%. In lymph nodes with a larger volume, metastatic tumor usually completely replaced the lymph node parenchyma. Thus, we consider the volume of inguinal lymph nodes in sarcoma 37 bearing mice a satisfactory and convenient surrogate marker for tumor metastasis assessment.

The total volume of bilateral regional ipsilateral plus contralateral lymph nodes was measured in mice bearing transplanted sarcoma 37. There are two limitations of such a nodes volume-based approach. First, its sensitivity is insufficient to detect early stages of metastasis. Second, the size of the affected lymph nodes does not always correspond to the amount of metastatic tumor cells because of possible reactive changes. Nevertheless, changes in the mean volume of mouse inguinal lymph nodes in the experimental groups directly and strongly correlated with primary S37 tumor growth and lifespan of animals in all our experiments.

**Figure 4:**
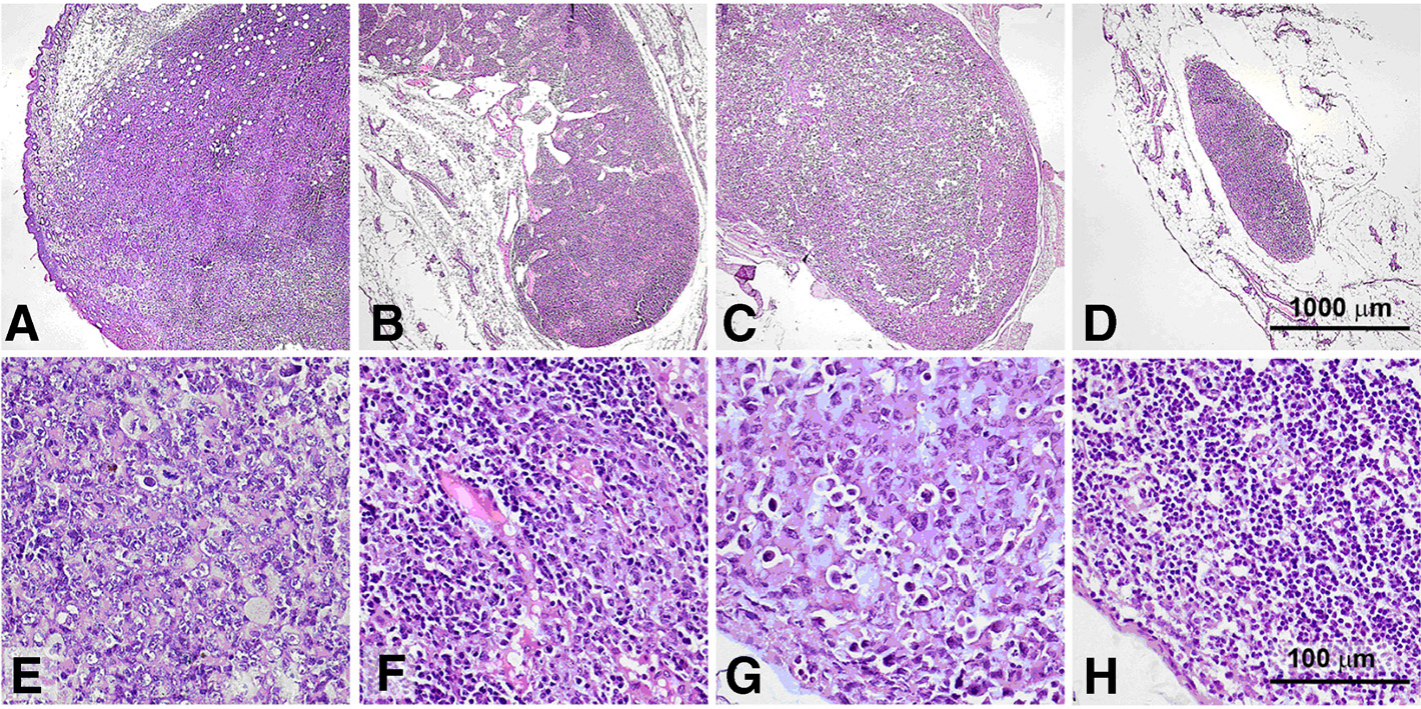
**Histological images of tumors and lymph nodes.** Subcutaneously transplanted mouse sarcoma 37 on day 15 of tumor growth **(a,d)**. Metastatic ipsilateral inguinal lymph nodes on the day 30 of tumor growth in control mice **(b,c,f,g)**. Images show tumor cells infiltrating lymph node parenchyma **(f)** and totally replacing lymph node tissue **(g)**. A lymph node taken on day 30 after the beginning of the treatment from a mouse treated with TKmGM/GCV **(h)**; note that its parenchyma is free of tumor cells. The sections are made through the largest cross dimension of the tissue samples. Low-power field images (a-d, 40×) demonstrate differences in size between positive (metastatic) and negative (metastasis free) lymph nodes. High-power field images (e-h, 400×) represent detailed histological features of the specimens. Formalin fixed and paraffin embedded tissues (H&E staining).

The bilateral inguinal lymph nodes of each mouse bearing sarcoma 37 were histologically examined to reveal metastatic involvement at the end of the surveillance period. Histological structure of the primary tumor and typical histological findings in the tissues of dissected lymph nodes are represented in Figure 4 (see a-c, e-g).

It was shown that the volume of lymph nodes in the groups of animals that received TKmGM, TK and mGM polyplexes combined with GCV was much smaller than that in the control group or the comparison groups (Table 1B). The highest metastasis inhibition (82%) was observed in mice that received TKmGM/GCV, whereas in the case of TK/GCV and mGM/GCV this inhibition was 67 and 66%, respectively. In the groups of animals treated with the constructs without GCV, metastasis inhibition was observed only for the TKmGM and mGM constructs (47 and 38%, respectively). This is in line with literature data on the essential role of GM-CSF in metastasis inhibition [41,64]. The data obtained demonstrated that the most efficient metastasis inhibition (high MI and low FLM) could be achieved with a combination of the *HSVtk* and *GM-CSF* genes within one construct.

### Estimation of the *in vivo* antitumor effect of the TKmGM-PPT/GCV system in different tumors

The therapeutic potential of the constructs in combination with GCV was evaluated in allograft mouse models inoculated with C26 (murine colon adenocarcinoma) and CSC5 (cervical squamous carcinoma) tumors.

Carcinoma CSC5 tumors were subcutaneously inoculated into F1 (С57Bl/6j x CBA) mice, and adenocarcinoma of the mouse large intestine (С26) was also subcutaneously inoculated into BALB/c mice. The treatment was started on day 7 of tumor growth, when the mean volume of tumor was about 100 mm^3^. The constructs were injected directly into tumors at a dose of 0.04 μg DNA/mm^3^ three times with 5-day intervals. GCV was administered for 15 days as intravenous infusions twice a day at a daily dose of 100 mg/kg for F_1_ mice and 150 mg/kg for BALB/c mice.

**Figure 5:**
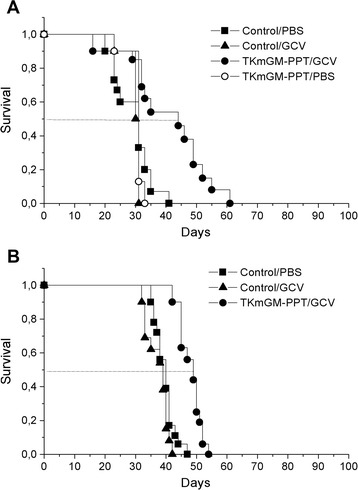
**Survival period of mice inoculated with A) adenocarcinoma C26; B) cervical squamous carcinoma CSC5 after injection of TKmGM-PPT.** TKmGM (CMV-HSVtk-mGM-CSF-pGL3 construct); PPT - polyethylenimine-polyethylene glycol-TAT peptide copolymer; PBS – phosphate buffered saline (placebo); GCV – ganciclovir.

The data obtained showed (Table 2, Figure 5) that treatment with TKmGM-PPT plus GCV had a biologically significant antitumor effect in animals with C26 tumors. On day 26 in the case of C26 ILS was 42%. TGD_1500_ in the animals with С26 was 8.5 days. In the case of CSC5 we observed TGD_1000_ equal to 6 days and a modest or even biologically insignificant extension of animal lifespan (23%). It may be due to the specific properties of CSC5 tumor or to a non-optimal scheme of treatment for this type of cancer. Injection of constructs without GCV or solely GCV did not appreciably affect tumor growth.

**Table 2:** **Effect of administration of the TKmGM-PPT complex on mice inoculated with C26 and CSC5 tumors**

**Complex or control solution**	**C26**		**CSC5**	
	**ILS%**	**TGD**_**1500**_ **days**	**ILS%**	**TGD**_**1000**_ **days**
TKmGM-PPT/GCV	42	8.5	20	6.0
TKmGM-PPT/PBS	−4	−0.3	-	-
Control/GCV	−12	0.8	0	−0.1
Control/PBS	-	-	-	-

C26 – female BALB/c mice with C26 tumor (groups of 10 animals), CSC5 – female BDF1 mice with CSC5 tumor (groups of 18 animals). Control – the group that received only GCV or PBS. The complexes were administered intratumorally 3 times in a single dose of 0.04 μg DNA/mm^3^ of tumor volume with a 5-day interval. The first administration was on day 7 of tumor growth. GCV – ganciclovir; GCV was administered for 15 days intraperitoneally twice a day with an interval of 12 h in a daily dose of 150 mg/kg (total dose of 2.25 g/kg). ILS –increase in lifespan of mice, TGD –tumor growth delay, MI –metastasis inhibition, mean values, FLM – frequency of lymphogenic metastasis (percentage of animals with metastases in lymph nodes). ILS and MI were measured on day 30 of tumor growth. PBS was administered in volumes equivalent to the GCV administration scheme.

Thus, treatment of sarcoma 37-bearing mice with TKmGM-PPT/GCV according to the scheme used is effective with respect to both the primary tumor focus (TGD 14.1 days) and metastasis (MI 82%, at the criterion of 35%, FLM 33%) (Table 1, Figure 3). These effects extended the lifespan of mice by 70% (at the criterion of 35%), and the biologically significant antitumor effect persisted for 14 days (at the criterion not less than 7 days). We have also shown that TKmGM-PPT/GCV is efficient for treatment of transplantable tumors C26 and almost ineffective for CSC5. Thus, the efficacy of TKmGM depends on the histological form of the tumor and declines in the row S37 > C26 > CSC5.

Earlier, it was shown that higher GCV doses enhance therapeutic efficacy of the HSVtk/GCV system [66]. Therefore, we have chosen the highest possible daily dose of GCV (150 mg/kg), and TKmGM-PPT was tested in allograft animal models at the background of GCV injection at a daily dose of 150 mg/kg for 15 days (total dose of 2.25 g/kg).

## Discussion

We studied the efficacy of the HSVtk/GM-CSF combination in one vector delivered by a nonviral carrier – a PEG-PEI-TAT copolymer. This combination was tested *in vitro* in various mouse and human cell lines, *ex vivo*, and *in vivo* using mouse models. It appeared to be efficient in terms of both tumor shrinkage and metastasis inhibition. The question is whether these results are promising for their further use in clinical practice. The problem in that preclinical efficacy of most drugs in mouse models is not the guarantee of positive clinical response is now widely debated. Even among cancer drugs that have passed Phase I testing, only 1 of 10 was finally approved [67]. Therefore, extrapolation of the results for these models to human disease is often not straightforward [67,68].

We will try to consider this problem from the standpoint of gene therapy prospects with respect to clinical trials.

### HSVtk can be active in human tumors providing proper delivery

HSVtk seems to be a very attractive potential drug due to its universal mechanisms of action. It is targeted at the cell’s replication machinery, which exists in all species, thus making HSVtk applicable to any tumor. Recent reports on preclinical cancer models demonstrated a high potential of HSVtk when used in combination with new therapeutic approaches [1]. One of the HSVtk great advantages is the bystander effect that allows to destroy not only cells transfected with the gene but also nearby untransfected cells, thus strongly increasing the potential efficacy [22,69,70]. Another potential advantage of HSVtk is its ability to stimulate the immune system to eliminate tumor cells that do not express the suicide gene, a phenomenon which could lead to the destruction of metastases originated from a primary tumor [19]. All these effects are not expected to be species specific. Indeed, we demonstrated that the *HSVtk* gene was translated into a functional HSVtk enzyme at a high level in human cells. Due to the advantageous features of *HSVtk*, the HSVtk/GCV was the first GDEPT system described. A large number of experiments were performed with this system in different types of tumors, and initial results in animal models were very promising [71]. However, the clinical trials were not so convincing, although they are still going on [1,17,19,71-73]. The barriers on the way of translation of preclinical models to clinical outcomes are most probably due to specific properties of model animal tumors and their microenvironment [74,75]. Apart from other aspects of intra- and inter-tumor variability, many authors pay great attention to a so called enhanced permeability and retention (EPR) phenomenon [4,76-81]. EPR is a property of well-developed and poorly differentiated solid tumor vasculature, the enhanced permeability of which allows nanoparticles of the size of up to several hundreds of nanometers to enter the tumor interstitial space, whereas the suppressed lymphatic filtration allows them to stay there. Now it is becoming clear that the EPR effect is much stronger in mouse tumors than in human patients, simply because most rodent tumors grow much faster. This directly concerns the HSVtk/GCV system which forms nanoparticles both in case of its delivery by viral and nonviral particles.

Moreover, penetration of drugs into the tumor and metastatic cells is hindered due to the properties of the tumor microenvironment. Hence, drug uptake can be different in animal and human tumors. Other peculiarities of intratumoral barriers and the biodistribution of drugs were considered in detail in the above cited reviews.

These highly complicated multilayer systems of barriers on the way of a drug to tumor cells stimulate the search for additional means to make tumor and its metastases more vulnerable to therapy. One of such means is activators of the host immune system, such as cytokines [82-84]. We used GM-CSF for this goal.

### GM-CSF is a potent immunomodulator both in mice and human: positive practice of its clinical application

Cytokines are used worldwide in clinical trials for the purposes of cancer gene therapy. The database [85] contains 1264 records devoted to cancer gene therapy clinical trials. 367 (29%) of them used cytokine genes as therapeutic agents, and 84 utilized GM-CSF. Data on synergistic activity of the suicide gene *HSVtk* and the immunomodulator GM-CSF support the GM-CSF capacity to enhance therapeutic effect [42]. There is a general belief that although local tumor control is important, efficient therapies to increase survival rate must also target metastases. Clinically, most cancer patients die of relapse and metastasis, and not of the primary tumor. However, the therapeutics capable of effectively preventing and targeting metastasis are very limited [86,87]. GM-CSF is considered one of the most effective inducers of tumor immunity [88]. It was reported to be able to enhance most types of immune responses [30]. The GM-CSF recombinant protein (Sargramostim/Leukine) was approved by FDA for clinical use. GM-CSF is extensively used in genetic engineering of (re-)infused cells for vaccination purposes [37].

Many oncolytic viruses armed with GM-CSF are now in clinical trials (see e.g. [26,32-35,54,89,90]). The data available permit to hope that GM-CSF will be as effective in cancer patients as in our model systems.

### Positive application of oncolytic viruses producing GM-CSF

Large tumor volume, cancer and stromal cells differently organized in different locations of the same tumor, and other negative factors [10,91-96] may create difficulties for both suicide and oncolytic virus therapy. This is an important reason for attempting to reach systemic efficacy by recruiting the immune system, instead of relying on oncolysis alone [90].

It has been already reported that several species of oncolytic viruses producing GM-CSF were tested in clinical trials and demonstrated safety, antitumor efficacy, and tumor-specific immune activation [54,90]. It was also shown that oncolytic virus therapies with GM-CSF expressing vectors can induce durable responses even in late stage solid tumors through the direct oncolysis and induction of anticancer immunity [33,34].

A hypothesis was put forward that tumor antigens released from dying cancer cells are accessible to antigen presenting cells which can activate cytotoxic T-lymphocytes for killing non-infected tumor cells [19,41,90]. This immune activation can be increased by locally produced GM-CSF, leading eventually to more aggressive antitumor immune response [90]. In case of suicide gene therapy, this hypothesis predicts that a combination of a suicide gene and GM-CSF within one vector will be preferable over a combination of two independent vectors each carrying only one of these two genes. The latter combination was tested earlier [41].

### Advantages of nonviral delivery systems

We used PEI-containing polyplexes modified with polyethylene glycol (PEG) and the cell-penetrating peptide TAT. The properties and behavior of these polyplexes in different cell lines could be adjusted to achieve higher transfection efficiency [57]. A detailed discussion of advantages and disadvantages of viral and nonviral delivery systems in gene therapy can be found in recent reviews [1,18,19,97-103]. Briefly, nonviral vectors are advantageous due to low immunogenicity, practically unlimited packaging capacity for genetic material, and simple and low-cost production, which makes them more suitable to large-scale production and potentially safer in clinical use [56,98,104-106]. However, they are characterized by low gene transfer efficiency and transient or steadily declining gene expression. On the other hand, viral vectors are characterized by low packaging capacity, relatively high production costs and a toxicity profile that can provoke inflammation and immunogenicity. Potential dangers of viral vectors to patients, staff, and possible shedding into the environment have resulted in rather stringent terms of use and risk assessments. Commercial manufacturing of therapeutic viruses, which is the final goal of research efforts, may meet serious problems and be highly expensive. These features have led to questioning the viability of virus-based approaches [55].

Keeping this in mind, it is interesting to analyze the latest data on GDEPT systems which are now in clinical trials according to the database “Gene therapy. Clinical trials worldwide” provided by the Journal of Gene Medicine [85], updated by July 2013.

According to the database, there are 9 Phase III, 12 Phase II and 91 Phase I ongoing clinical trials for treatment of various cancers with suicide genes in various vectors. Adenoviral, retroviral and herpes simplex type 1 viral vectors were used in 64, 66, and 11 trials, respectively. Only a few trials used inexpensive delivery systems: 5 trials used naked plasmid DNA and two trials - lipofection.

Such an evident “viral bias” is in line with the opinion expressed by Duenas-Gonzalez and colleagues: “The high-risk/high-reward aspect of drug discovery comprises a greater issue in the commercial realm in terms of new-compound approval and marketability. Therefore, oncological products are subject to the laws of marketing; hence, the majority of the newer cancer products are simply cost-prohibitive to the vast majority of patients worldwide, which has been widely approached and reviewed” [107].

## Conclusions

Apart from efficacy and safety, it is desirable that each drug should be inexpensive and affordable to a wide range of patients. In present-day GDEPT clinical trials, viral delivery systems dominate due to efficient transfection and production of therapeutic genes. However, safety concerns associated with immune and inflammatory responses as well as high cost of therapeutic viruses can limit their wide therapeutic use. In this regard, nonviral delivery systems seem to be advantageous despite lower efficiency. An important attribute of suicide therapy is the so-called bystander effect, which can partially compensate for the poor efficiency due to the diffusion of intracellular toxin to neighboring tumor cells. A further increase in the efficiency of GDEPT can be achieved by induction of antitumor immune response which should also lead to the destruction of metastases.

Therefore, we designed and experimentally tested nonviral vectors that carried both the suicide (HSVtk) and immunomodulating (GM-CSF) genes encapsulated inside the envelope made of a PEG-PEI-TAT copolymer. The vectors were administered intratumorally. We expected a synergistic effect of the two therapeutic genes because of a high local concentration of both tumor antigens from destroyed cancer cells and the cytokine recruiting immune antigen-presenting cells. In fact, we found that the simultaneous intracellular expression of the HSVtk and GM-CSF genes had a greater antitumor effect than the expression of HSVtk or GM-CSF alone. In particular, the treatment of animals with the TKmGM-PPT system was, in most cases, able to inhibit the growth of tumor and metastases and increase lifespan. Thus, the data obtained here show that quite efficient antitumor effects can be achieved using a simple and low toxic nonviral carrier – a PEG-PEI copolymer, providing that the vector contains both the suicide and immunomodulating gene. Study of the TKmGM-PPT toxicity demonstrated safety of this complex. The observed toxic reactions in animals were weak and fully reversible (to be published elsewhere). We believe that the results obtained are promising enough to move towards clinical trials.

Vector constructions used in the work: TKmGM – CMV-HSVtk-mGM-CSF-pGL3 construct, TKhGM – CMV-HSVtk-hGM-CSF-pGL3 construct, TK – CMV-HSVtk-pGL3 construct, hGM – CMV-hGM-CSF-pGL3 construct, mGM – CMV-mGM-CSF-pGL3 construct, TKGM – collective designation for CMV-HSVtk-mGM-CSF-pGL3 and CMV-HSVtk-hGM-CSF-pGL3 construct.

## Additional files

Additional file 1: Figure S1. Production level of HSVtk expressed from the TK and TKmGM constructs transfected with LFA. Additional file 2: Figure S2. Biological activity of HSVtk expressed from the TK and TKmGM constructs transfected with LFA. Additional file 3: Table S1.
*In vitro* cytotoxic effect of TKGM complexed with PPT or LFA, in combination with GCV. Additional file 4: Table S2.
*In vivo* cytotoxic effect of TK, mGM or TKmGM complexed with PPT or LFA in combination with or without ganciclovir. Additional file 5: Figure S3. Survival period of sarcoma 37-bearing mice after injection of TK-LFA, mGM-LFA and TKmGM-LFA with or without ganciclovir.

## Abbreviations

5-FC: 5-fluorocytosine
5-FU: 5-fluorouracil
CD: Cytosine deaminase
EPR: Enhanced permeability and retention effect
FLM: Frequency of lymphogenic metastasis
GCV: Ganciclovir
GDEPT: Gene directed enzyme prodrug gene therapy
GM-CSF: Granulocyte–macrophage colony-stimulating factor
hGM-CSF: Human GM-CSF
mGM-CSF: Mouse GM-CSF
HSVtk: Herpes simplex virus thymidine kinase
IFN-γ: Interferon γ
IL: Interleukin
ILS: Increase in lifespan
IRES: Internal ribosome entry site
LFA: Lipofectamine
MI: Metastasis process inhibition
PBS: Phosphate buffered saline
PEG: Polyethylene glycol
PEI: Polyethylenimine
PPT: Polyethylenimine-polyethylene glycol-TAT peptide copolymer
TGD: Tumor growth delay
TNF-α: Tumor necrosis factor α
TTE: Time to endpoint
