# Supplementary material for: Therapeutic properties of a vector carrying the HSV thymidine kinase and GM-CSF genes and delivered as a complex with a cationic copolymer
Source: J Transl Med. 2015 Mar 4;13:78. doi: 10.1186/s12967-015-0433-0 (PMC4359447; doi:10.1186/s12967-015-0433-0)
Supplement: Additional file 1: Figure S1. — Production level of HSVtk expressed from the TK and TKmGM constructs transfected with LFA. [file 12967_2015_433_MOESM1_ESM.pdf]

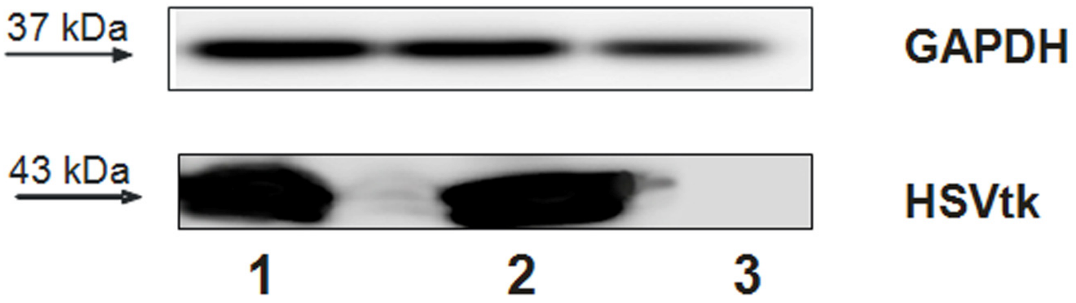

Figure S1. Production level of HSVtk expressed from the TK and TKmGM constructs transfected with LFA. Immunoelectrophoretic staining of the HSVtk and GAPDH proteins from HEK293 cells transfected with the TK (1) or TKhGM (2) constructs, 3 – non-transfected cells.
