# Supplementary material for: Therapeutic properties of a vector carrying the HSV thymidine kinase and GM-CSF genes and delivered as a complex with a cationic copolymer
Source: J Transl Med. 2015 Mar 4;13:78. doi: 10.1186/s12967-015-0433-0 (PMC4359447; doi:10.1186/s12967-015-0433-0)
Supplement: Additional file 2: Figure S2. — Biological activity of HSVtk expressed from the TK and TKmGM constructs transfected with LFA. [file 12967_2015_433_MOESM2_ESM.pdf]

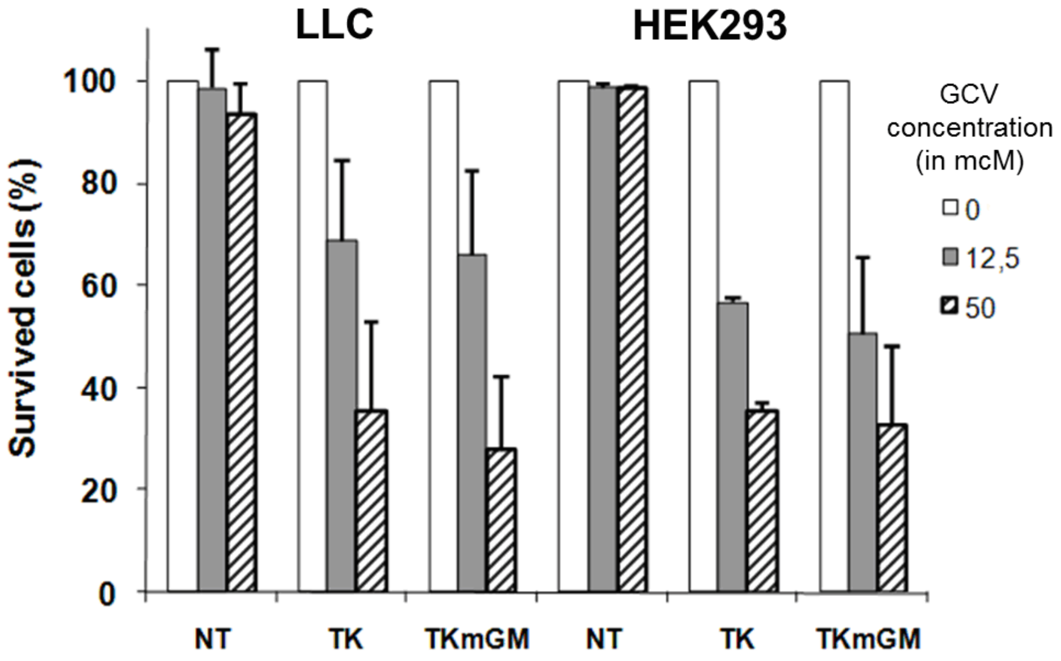


**Figure S2**. **Biological activity of HSVtk expressed from the TK and TKmGM constructs transfected with LFA.** Survival of HEK293 and LLC cells transfected with the TK and TKmGM constructs in the presence of GCV. The height of histogram bars corresponds to the percentage of survived LLC and HEK293 cells in 196 h after addition of GCV. On the right – concentration of ganciclovir. NT – non-transfected cells; TK – cells transfected with the CMV-HSVtk-pGL3 construct; TKmGM – cells transfected with the CMV-HSVtk-mGM-CSF-pGL3 construct.
