# Supplementary material for: Therapeutic properties of a vector carrying the HSV thymidine kinase and GM-CSF genes and delivered as a complex with a cationic copolymer
Source: J Transl Med. 2015 Mar 4;13:78. doi: 10.1186/s12967-015-0433-0 (PMC4359447; doi:10.1186/s12967-015-0433-0)
Supplement: Additional file 3: Table S1. — In vitro cytotoxic effect of TKGM complexed with PPT or LFA, in combination with GCV. [file 12967_2015_433_MOESM3_ESM.pdf]

Table S1. *In vitro* cytotoxic effect of TKGM complexed with PPT or LFA, in combination with GCV.

**GCV, mcM**	2	12,5	50	200	2	12,5	50	200	2	12,5	50	200	2	12,5	50	200
	**Percentage of survived cells**															
**Cell line**	**S37**				**C26**				**A431**				**HT1080**			
**TKGM-PPT**	89	72	34	7	62	37	26	7	97	90	72	47	97	87	69	53
**TKGM-LFA**	***100***	62	25	7	14	9	7	3	76	54	41	30	60	35	23	15
**NT**	***100***	100	***100***	48	100	***100***	99	24	***100***	***100***	98	71	***100***	100	97	91

Mouse tumor cells S37 and C26 were transfected with TKmGM complexed with either PPT or LFA; human tumor cells A431 and HT1080 were transfected with TKhGM complexed with either PPT or LFA. NT – non-transfected cells. The transfected and control cells were incubated for 4 days with different concentrations of ganciclovir (GCV). The data are presented as MTS test results in percentage of survived cells as compared with the control (the number of cells survived in medium lacking GCV). The values over 100% (102-125%) are shown in italics. The spread of values was 0-4%.
