# Supplementary material for: Therapeutic properties of a vector carrying the HSV thymidine kinase and GM-CSF genes and delivered as a complex with a cationic copolymer
Source: J Transl Med. 2015 Mar 4;13:78. doi: 10.1186/s12967-015-0433-0 (PMC4359447; doi:10.1186/s12967-015-0433-0)
Supplement: Additional file 4: Table S2. — In vivo cytotoxic effect of TK, mGM or TKmGM complexed with PPT or LFA in combination with or without ganciclovir. [file 12967_2015_433_MOESM4_ESM.pdf]

Table S2. *In vivo* cytotoxic effect of TK, mGM and TKmGM complexed with PPT or LFA in combination with or without ganciclovir.

Constructs and control	Lifespan days	TGD_{500} days	FLM %	Volume of lymph nodes, mm^3	MI, %
TKmGM-LFA/GCV	59±13	12.4	42*	108±72	81*
TK-LFA/GCV	59±24	12.7	67	167±51	70*
mGM-LFA/GCV	37±9	3.2	33*	261±79	54
TKmGM-PPT/GCV	60±22	14.1	33*	100±45	82*
TK-PPT/GCV	57±15	11.6	58*	186±80	67*
mGM-PPT/GCV	39±13	4.1	50*	189±91	66
TKmGM-LFA	37±3	4.4	50*	318±98	44
TK-LFA	37±4	4.3	83	452±112	20
mGM- LFA	47±6	4.2	58*	290±142	49
TKmGM-PPT/PBS	41±7	3.9	42*	298±264	47
TK-PPT/PBS	38±4	2.6	75	459±339	18
mGM-PPT/PBS	48±4	3.0	58*	349±242	38
Control/GCV	40±3	0.3	100	457±121	19
Control/PBS	35±3		100	562±316	

F1 (C57Bl/6jxCBA) female mice (12 animals in each group) were inoculated with sarcoma 37 on day zero. TKmGM (CMV-HSVtk-mGM-CSF-pGL3 construct), TK (CMV-HSVtk-pGL3), mGM (CMV-mGM-CSF-pGL3); PBS – phosphate buffer saline (placebo); GCV – ganciclovir, PPT - polyethylenimine-polyethylene glycol-TAT peptide copolymer, LFA – Lipofectamine-2000. Control – the group that received only GCV or PBS; TGD_{500} –tumor growth delay, MI – metastasis process inhibition, mean values, FLM – frequency of lymphogenic metastasis (percentage of animals with metastases in lymph nodes). ILS, MI (compared with the control group of animals that received PBS) and the volume of lymph nodes were measured on day 30 after inoculation. The constructs and control solutions were administered intratumorally with a 5-day interval between administrations. PBS was administered in volumes equivalent to the GCV administration scheme. The PPT concentration in injected solutions of the constructs was 25 µM

* - statistically significant values ($p<0.05$).
