# Supplementary material for: Therapeutic properties of a vector carrying the HSV thymidine kinase and GM-CSF genes and delivered as a complex with a cationic copolymer
Source: J Transl Med. 2015 Mar 4;13:78. doi: 10.1186/s12967-015-0433-0 (PMC4359447; doi:10.1186/s12967-015-0433-0)
Supplement: Additional file 5: Figure S3. — Survival period of sarcoma 37-bearing mice after injection of TK-LFA, mGM-LFA and TKmGM-LFA with or without ganciclovir. [file 12967_2015_433_MOESM5_ESM.pdf]

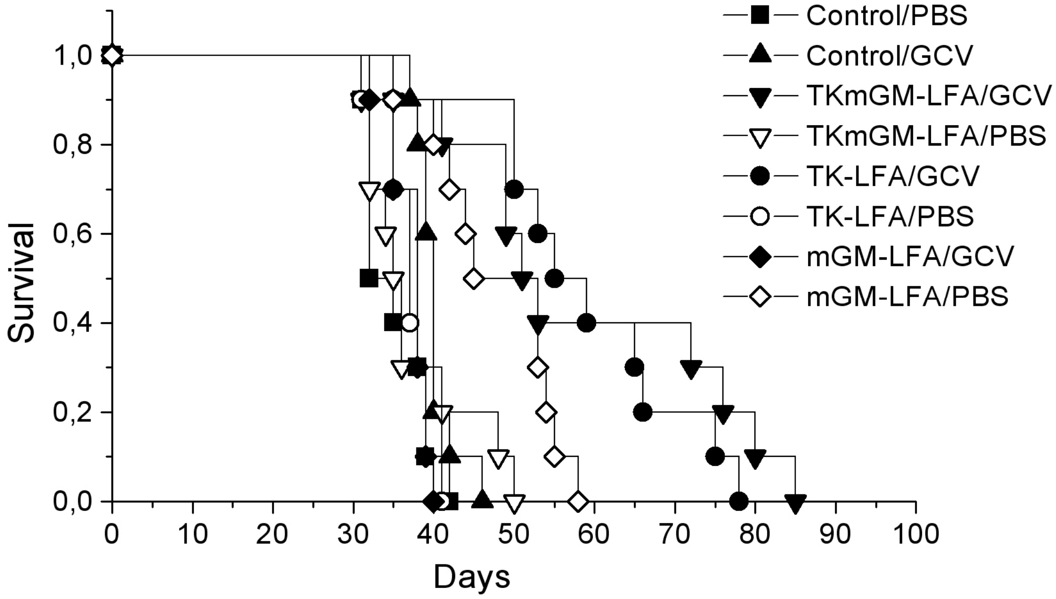

Figure S3. **Survival period of sarcoma 37-bearing mice after injection of TK-LFA, mGM-LFA and TKmGM-LFA with or without ganciclovir.** TKmGM (CMV-HSVtk-mGM-CSF-pGL3 construct), TK (CMV-HSVtk-pGL3), mGM (CMVmGM-CSF-pGL3); PBS – phosphate buffered saline (placebo); GCV – ganciclovir, LFA – lipofectamine.
